# Ablation of the sacroiliac joint using MR-guided high intensity focused ultrasound: a preliminary experiment in a swine model

**DOI:** 10.1186/s40349-017-0095-x

**Published:** 2017-06-26

**Authors:** Elena A. Kaye, Majid Maybody, Sebastien Monette, Stephen B. Solomon, Amitabh Gulati

**Affiliations:** 1 Department of Medical Physics, Memorial Sloan Kettering Cancer Center, 1275 York Ave, New York, NY 10065 USA; 2 Department of Radiology, Memorial Sloan Kettering Cancer Center, 1275 York Ave, New York, NY 10065 USA; 3 Tri-Institutional Laboratory of Comparative Pathology, Memorial Sloan Kettering Cancer Center, The Rockefeller University, Weill Cornell Medical College, 1275 York Ave, New York, NY 10065 USA; 4 Department of Anesthesiology-Critical Care, Memorial Sloan Kettering Cancer Center, 1275 York Ave, New York, NY 10065 USA

**Keywords:** Sacroiliac joint, Back pain, Focused ultrasound, MRgHIFU, HIFU, FUS, Ablation, Nerve, MRI, Denervation, Neurolysis

## Abstract

**Background:**

Dysfunction of the Sacroiliac Joint (SIJ) is one of the key sources of low back pain. For prolonged pain relief, some patients undergo fluoroscopic guided radio-frequency (RF) ablation of SIJ, during which a number of RF probes are inserted to create thermal lesions that disrupt the posterior sacral nerve supply. This procedure is minimally invasive, laborious, time-consuming and costly. To study if High Intensity Focused Ultrasound (HIFU) ablation is a feasible alternative approach to SIJ pain treatment, we performed experiments using HIFU to ablate SIJ in the swine model.

**Methods:**

Three female Yorkshire swine (36, 35.2 and 34 kg) underwent bilateral Magnetic Resonance guided HIFU (MRgHIFU) ablation of the SIJs. Treatment assessment was performed using contrast-enhanced imaging, histopathology and evaluation of pain and changes in ambulation and gait.

**Results:**

Contiguous lesions along the right and left SIJs were achieved in all animals. In one out of three animals, excessive heating of the muscle and skin tissue in the near-field resulted in unwanted muscle necrosis. No changes in animal behavior, ambulation or gait were detected.

**Conclusions:**

The initial experiments with MRgHIFU ablation of SIJs in sub-acute swine model show promise for this ablation modality as a non invasive and more precise alternative to the currently used fluoroscopically - guided RF ablations and injections.

## Background

Dysfunction of the Sacroiliac Joint (SIJ) is a source of pain in approximately 15 to 30% of patients who suffer from low back pain [1, 2], a condition that carries a worldwide, significant public health burden and is among the top three causes of years lived with disability in the United States [3, 4].

The SIJ and the posterior sacroiliac ligament are reported to be innervated by the dorsal ramus of L5 and the lateral branches of S1 to S4 nerve roots [5–8]. These branches emerge from the posterior sacral foramina and vary in number and location relative to the foraminal apertures [5, 9]. Currently, for prolonged pain relief, patients may undergo intra-articular injection of local anesthetic and corticosteroid, and/or radio-frequency ablation (RFA) of the posterior sacral nerve supply of the joint [10, 11]. During the RFA of the SIJ, the RF probes are placed between the posterior sacral foramina and the SIJ. To create a contiguous ablation zone disrupting all the lateral branches innervating the SIJ. Up to 12 RF probes are inserted on each side of the sacrum, and ideally the probes are repositioned to create thermal lesions targeting the lateral branches at various distances from sacral foramina [12]. To avoid injuring the sacral ventral nerve roots emerging from the sacral canal via the anterior foramina, peri foraminal placement of the RF probes is carried out with caution under fluoroscopy guidance.

The evidence for the therapeutic effectiveness of RFA outweighs that of intra-articular injections [13, 14]. However, the large number of RF probes required to create a contiguous lesion and inability to image probe placement in real-time, due to ionizing radiation, make RFA denervation of the SIJ very laborious, time-consuming and costly [15].

Magnetic Resonance Imaging guided High Intensity Focused Ultrasound (MRgHIFU) is a unique ablation modality that can noninvasively create thermal lesions of any shape deep inside the body under a real-time temperature monitoring. In recent years, MRgHIFU has been found to be clinically effective for pain palliation of bone metastasis, osteoarthritis, osteoid osteoma and facet arthritis [16–21]. In preclinical model, MRgHIFU was applied to ablate the facet joint [22, 23], lumbar medial branch nerves [24], intercostal [25], sciatic [26, 27] and sympathetic renal nerves [28]. Based on its unique properties, MRgHIFU may be a promising modality for treatment of SIJ dysfunction in chronic low back pain. First, using MRgHIFU would not require insertion and repositioning of the numerous probes. Second, MR thermometry, used to monitor heating of tissue during a HIFU procedure, would make it possible to ensure continuity of a lesion during the procedure allowing for treatment modification to avoid incomplete ablation. Additionally, the on-going advancement of MRI neurography methods may make it possible to visualize the small branch nerves innervating the SIJ and thus improve the precision of the procedure.

The challenges of MRgHIFU ablation of the SIJ are the potential risk of damaging thermal exposure of the adjacent untargeted sacral nerves, sacral bone, adjacent muscle and skin. The ventral nerve roots may be harmed if ultrasound of sufficient intensity reaches directly through the posterior sacral foramina or heats the adjacent sacral bone, which in turn conducts the heat to the nerve roots. Proximity of skin to the targeted SIJ may expose the skin and adjacent muscle tissue to the pre-focal portion of the ultrasound beam, potentially causing unwanted heating. To study if HIFU ablation of the SIJ may be a feasible clinical procedure, we performed preliminary experiments using MRgHIFU to ablate the SIJ in three swine.

Unlike lumbar spine, where swine is considered a suitable animal model, the appropriateness of swine as an animal model for studying sacral ablation is not discussed in the literature. Hence, the first section of this study presents qualitative description of the selected swine animal model as it relates to the human sacral anatomy. The subsequent sections of the study present our experimental approach and the effects of the HIFU ablation as they appear on follow-up imaging, histology and behavioral assessment.

## Methods

All animal procedures received approval from the Institutional Animal Care and Use Committee. Appropriate handling and care was provided by trained staff in accordance with the principles of laboratory animal care and guidelines from the United States Department of Agriculture.

### Animal model

To evaluate if the swine model can approximate human sacral anatomy, veterinary anatomy literature was surveyed. The computerized tomography (CT) images of swine available in our laboratory were reviewed to observe the bony anatomy of porcine sacrum. Appearance of the porcine sacral nerves was described based on the histology images prepared as described in Treatment Assessment section.

### Animal protocol

Animal protocol used in this study is described in detail in [24]. Briefly, three female Yorkshire swine (weighing 36, 35.2 and 34 kg) underwent MRgHIFU ablation of the SIJs. Each animal was sedated and then anesthetized by subcutaneous injection of carprofen and intravenous injection of buprenorphine. Skin hair was removed from the sacral region and the animals were intubated. Clinical anesthesia was maintained using Isoflurane 2–3% with 100% oxygen on a Penlon Nuffield MRI-compatible ventilator (Penlon, Inc., Minnetonka, MN, USA). Heart rate and pulse oximetry were continuously monitored. Following the procedure, the animals were maintained on preventative medications for potential pain. Both pain and behavioral assessments, including evaluating ambulation and gait, were performed twice daily by the veterinary staff. Forty-eight hours after the procedure, the animals underwent follow-up imaging and were euthanized with an overdose of intravenous Euthasol while under anesthesia.

### MRgHIFU treatment

**Fig. 1:**
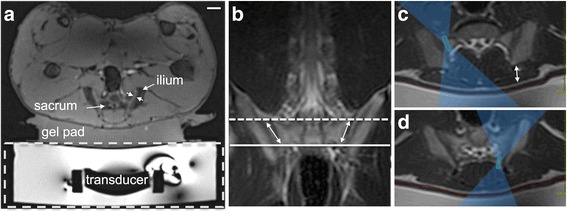
Experimental setup and treatment planning. **a**. The swine is positioned supine on top of a gel pad on the HIFU MRI table (dashed box) housing the HIFU transducer in a water bath. **b**. Coronal MR image of the swine showing the region of the sacrum where HIFU treatment was applied. The most cranial and caudal locations of the treatment are shown in dashed and solid lines, respectively. Arrows indicate the approximate targeted area. Two types of targeting approach are shown in **c** and **d**. Axial MR images (**c** corresponds to the dashed line, and **d** - to solid line in figure **b**) show the overlay of the HIFU beam and prescribed sonications used to treat the right (**c**) and left (**d**) SIJ

A clinical HIFU system (ExAblate 2000®; InSightec Ltd., Haifa, Israel) installed in a 3 Tesla MRI scanner (SIGNA; GE Healthcare, Waukesha, WI, USA) was used. The animals were placed on the MRgHIFU table in a supine, feet-first position. A polyacrylamide gel pad was used between the tissue and HIFU transducer bath to provide acoustic coupling (Fig. 1a). Planning MR images of the sacrum (slice thickness 3 mm or 4 mm) were acquired using a T2-weighted sequence and loaded into a treatment planning workstation (InSightec Ltd.). An experienced, board-certified pain medicine anesthesiologist (AG) prescribed the sonication positions medial to and directly on the SIJ. Sonications were planned in the slices between the slice superior to the S1 foramina and the most inferior slice depicting the SIJ (Fig. 1b). After placing the focal spot of each sonication using the axial plane, the angle of the ultrasound beam was adjusted in the supine plane to achieve near perpendicular incidence of the HIFU beam onto the surface of sacrum. In each animal, on the right side, the focal spot of a sonication was placed behind the bone-muscle boundary as commonly done during MRgHIFU treatment of painful bone metastases (Fig. 1c), and on the left side, the position of the sonications was prescribed immediately at the interface between bone and tissue (Fig. 1d). Anticipating larger area of ablation using the near-field targeting approach used on the right side, fewer sonications were prescribed compared to the focal targeting approach used on the left side.

The central frequency of focused ultrasound was increased to 1.35 MHz to minimize ultrasound penetration into bone tissue [22]. The focal distance (the distance from the surface of the transducer to the focal spot) ranged from 105 mm to 157 mm, depending on the weight of the animal and the position of the target. The length of the focal spot was between 11 mm and 12 mm. The duration of each HIFU application was 20 s, and the time between the consecutive sonications ranged from 1.5 and 4 min to allow for cooling of the skin and the muscle tissue between the skin and the targeted bone region. The temperature during each sonication was monitored in muscle adjacent to bone in near-real-time using proton-resonance-frequency-shift-based MRI thermometry. Single-slice temperature maps were acquired in axial, supine or coronal planes containing the center of sonication’s focal spot. The detailed treatment parameters are listed in Table 1.

**Table 1:** Summary of the in vivo experiments

	S1 Right	S1 Left	S2 Right	S2 Left	S3 Right	S3 Left
Number of sonications	8	37	4	8	5	12
Acoustic energy (J)	1163 ± 154	747 ± 90	835 ± 42	654 ± 0	689 ± 0	574 ± 0
Cooling time (s)	130 ± 47	133 ± 33	405 ± 177	474 ± 137	393 ± 160	424 ± 241
Focal height (mm)	151 ± 6	116 ± 8	-	-	123 ± 5	114 ± 5
Maximum Temperature (C)	71 ± 5	63 ± 5	75 ± 7	86 ± 6	85 ± 14	77 ± 10
MR thermometry imaging plane	Axial(8)	Axial(16) Sag (19) Cor(2)	Axial(3) Sag (1)	Sag (8)	Axial(3) Sag (2)	Sag (12)

### Treatment assessment

The contrast-enhanced MRI (CE-MRI) was performed immediately following the procedure, using intravenous gadopentetate dimeglumine MRI contrast agent (Magnevist®; Bayer HealthCare, Bayer AG, Leverkusen, Germany), 0.2 ml/kg (0.1 mmol/kg) and T1-weighted fast spoiled gradient-recalled echo imaging. Forty-eight hours after treatment, the animals were imaged on a Computerized Tomography (CT) scanner (Innova; GE Healthcare, Milwaukee, WI, USA) using iohexol contrast medium (Omnipaque 300; GE Healthcare, Milwaukee, WI, USA). On CE-MRI and CT images, the extent of thermal necrosis was inferred from the regions lacking contrast uptake. Twice a day, the veterinary staff visually inspected the skin for potential burns and observed the animals for altered gait, abnormal posture, changes and asymmetry in movement. Pain assessment was performed via observation and palpation, both superficial and deep [29].

Histopathological analysis of treated tissue was performed per the following steps. First, the whole sacrum, including the sacroiliac joints and muscle attached to the bone, were fixed by immersion in 10% neutral buffered formalin for approximately six weeks until complete fixation was achieved. Second, the specimen was placed into decalcifying solution Surgipath Decalcifier I (Leica Biosystems, Richmond, IL, USA) for eight to ten weeks. Upon complete decalcification, the sample was sectioned transversely into 5 mm thick slices and each slice was inspected for any visible tissue changes. Then the areas of interest in each slice were excised en bloc with adjacent muscle and bone. For each study respectively, thirty five, fourteen and sixteen histological samples (with 11, 4 and 8 sections containing the nerve roots, and 4 sections containing the spinal cord) were processed routinely in alcohol and xylene, embedded in paraffin, sectioned at 5-micron thickness, and stained with hematoxylin and eosin (H&E). Each histological sample was analyzed for changes in muscle, bone and neural tissue using a microscope (Olympus BX45) with objectives ranging from 1.25X to 60X. The histology slides from the second and third experiment were also scanned with a digital scanner (Pannoramic Flash 250, 3DHistech, Budapest, Hungary), using 20×/0.8NA objective. The digital scans of the slides were then analyzed using dedicated software (Pannoramic Viewer, 3DHistech, Budapest, Hungary). The dimensions of the nerves, vessels and the distances between various structures were measured using the software measuring tool. Based on the noted tissue changes, the region of ablation was delineated in several samples to compare to the apparent ablation zone as visible on gross pathology and imaging. Additionally, these histological samples were used to describe the innervation of the SIJ.

## Results

### Animal model

**Fig. 2:**
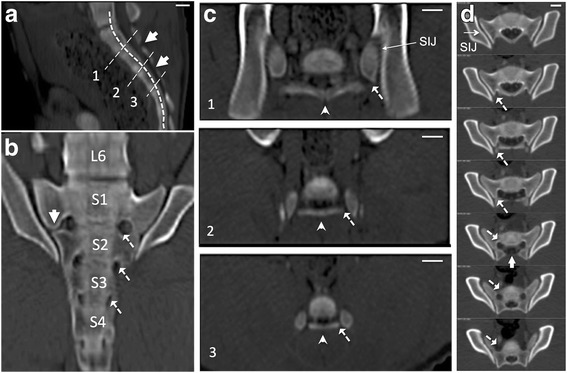
Porcine sacral anatomy as visualized on contrast-enhanced CT images. **a**) Sagittal section through the median line. Incomplete posterior fusion of the sacral vertebrae are shown with thick arrows. **b**) Curved planar reformation of the axial images along the curve shown with thick dashed line in **a**. The four sacral vertebrae are labeled and dashed arrows show three pairs of the posterior sacral foramina apertures. **c**) Oblique planar reformation images (obtained along the straight thin dashed lines in **a**) showing the entire sacral foramina (dashed arrows) including the dorsal and ventral sections. Arrowheads indicate the nearly absent spinous process. **d**) Unformatted axial CT images of the sacral region (more cranial slices are shown first) show the gap between S1 and S2 vertebrae (thick arrow) and S1-S2 sacral foramen (dashed arrows)

Sacral anatomy of swine was found similar to human. Some differences between the two are as follows. Compared to human sacrum, consisting of five vertebral bodies, in swine, sacrum consists of four vertebrae (Fig. 2a, b). They fuse later in life [30, 31] (Fig. 2b). The dorsal surface, which is flattened and smooth, presents openings into the sacral canal between the adjacent arches [32] (Fig. 2a, d). In swine, sacrum is triangular as in humans [33], however, the vertebrae are narrower. While the spinous processes are pronounced in human sacrum, in swine they are nearly absent [31, 32] (Fig. 2c). The dorsal branch of the nerve root continues from the sacral canal in the outward and caudal direction exiting the smaller dorsal foramen. The dorsal foramen (Fig. 2c) is lateral and caudal to its corresponding ventral foramen and therefore, these foramina are not visible in their entirety in one single axial image (Fig. 2d). The diameter of the foramina in swine used in this study were found to be equal or less than 5 mm. Additionally the SIJs are tilted ventrally making the ventral distance between the two joints longer than the dorsal distance. Therefore, in the swine used here, the distance between the posterior foramina and the SIJ, 5 mm or less, is much smaller than that in human.

The spinal cord, that normally terminates at the lumbar vertebrae level in humans, extends to the S2–S3 level in pigs [34]. No reports of the SIJ innervation in swine were found. Examining the transverse sections of gross pathology and histology of the L6 to S3 specimens used here, the spinal cord and the nerve roots were visible in the sacral canal at each level. Two large vessels of approximately 2–5 mm in diameter were also present in the sacral canal.

**Fig. 3:**
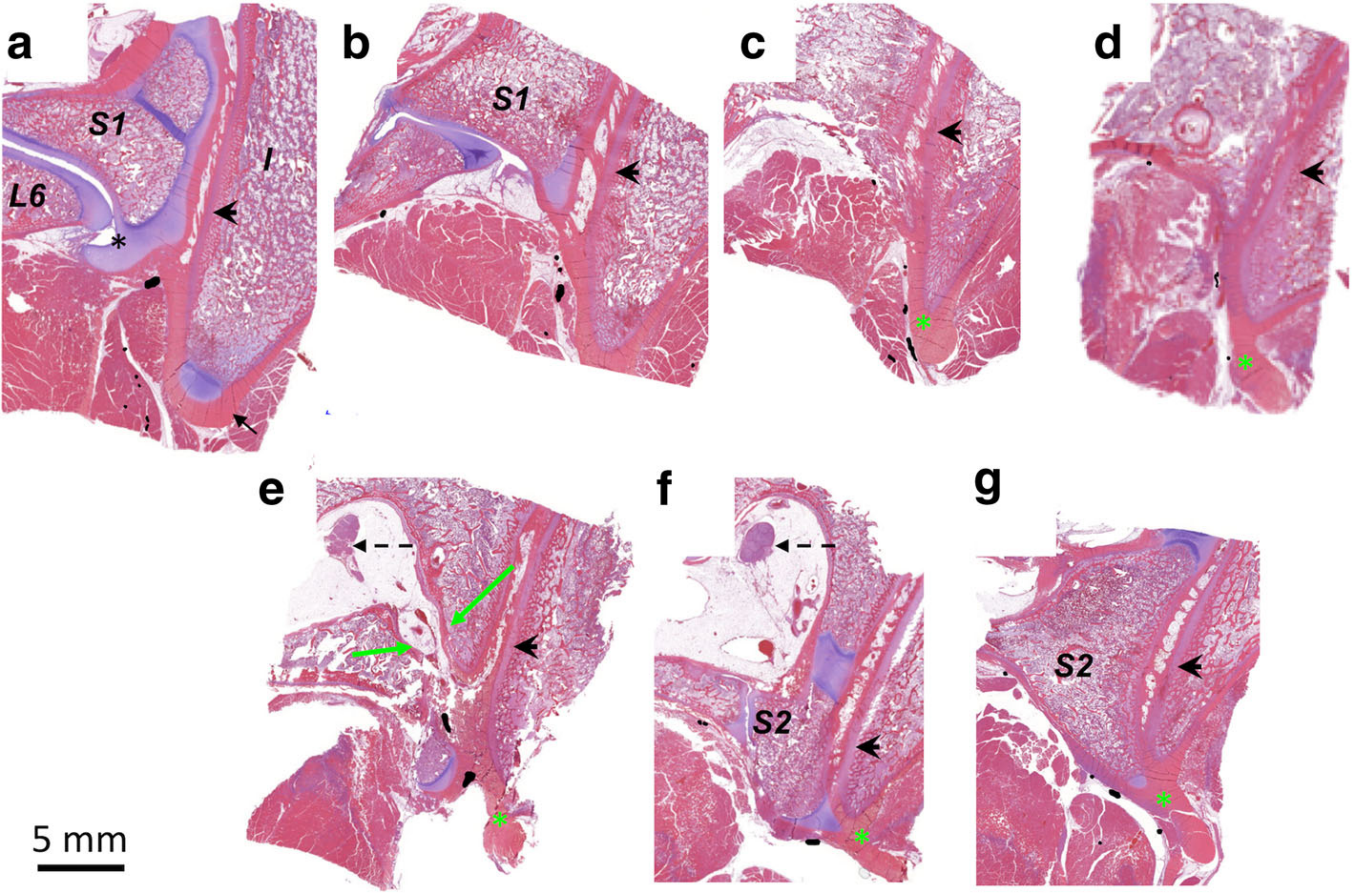
Innervation of the SIJ in a swine. (**a**-**g**) Nerves (black color), contoured using 40× magnification, are visible in every section with the largest diameter branches in A and E. Vertebral lumbar and sacral bodies and ilium are labelled with *L6,S1,S2* and *I* (ilium). (**a**) Cartilage (black asterisk) and ligament (black arrow) are marked. (**c**-**g**) Green asterisk indicates the tip of the ligament (long ligament). (**a**-**g**) SIJ is marked with black arrowhead. (**e**) S1-S2 dorsal foramen is shown with green arrows and the anterior sacral nerve roots are shown with dashed arrows. Scale bar is 5 mm

Histologic analysis of the sections of L6-S1, S2 and S3 vertebral bodies (Fig. 3) showed that posterior branches of lumbar dorsal ramus and lateral branches of the sacral dorsal nerve roots first travel posteriorly along the surface of the sacrum and SIJ (Fig. 3a-b, e-f) and then posterior-caudally along the surface of the posterior superior iliac spine (Fig. 3c-d, f-g). Up to 1 mm thick layer of ligament tissue was found between the bone and muscle tissue. In addition to the larger posterior branch nerves ranging in diameter from 1 mm down to few hundred microns, smaller nerves (with diameter less than 100 microns) were found in muscle tissue and along the dorsal surface of the sacral spine. The largest diameter nerves were found in the slices containing or adjacent to the L6-S1 joint and sacral dorsal foramina (Fig. 3a, e). The diameter of the nerves reduced in caudal direction. The distance between the posterior branch nerves and bone (covered with ligament) or the ligament ranged from 0 to 500 microns. Measured for 30 larger nerves, the distance between the nerve and the closest vessel ranged from 0 to 2 mm with average distance of 315 microns. The diameter of the vessels ranged from 40 to 400 microns, with average diameter of 130 microns.

### Treatment assessment

**Fig. 4:**
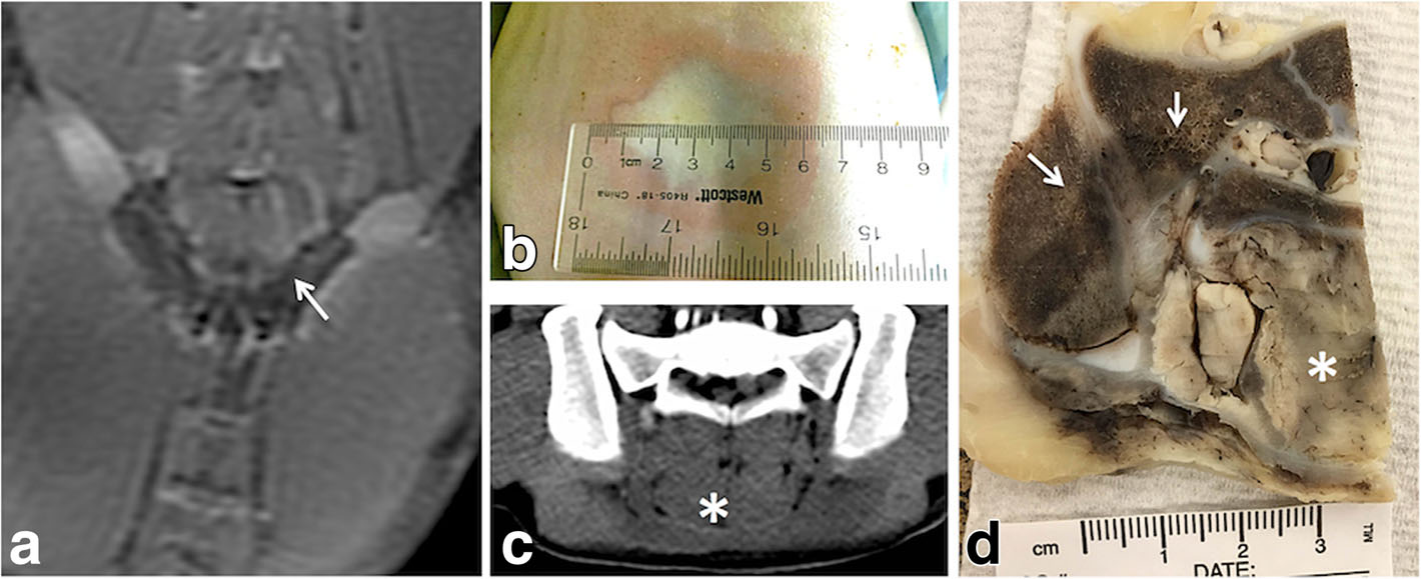
**a**. Contrast-enhanced coronal MR image showing the contiguous elongated regions of perfusion loss positioned along the left and right SIJ, indicating ablation zone. **b**. Skin changes observed Immediately after the treatment. Unplanned extent of ablation zone (asterisk) in muscle achieved in experiment 1 is shown on contrast-enhanced axial CT image (**c**) and gross-pathology (**d**)

Contrast-enhanced MR and CT imaging, gross pathology and histology showed that contiguous lesions along the right and left SIJs were achieved in all animals (Fig. 4a). In the first animal experiment, a white region surrounded by a pink contour was observed on the skin of the animal (Fig. 4b). The diameter of this region reduced from 6 cm as measured immediately following the treatment to 1 cm - 48 h after the treatment. Additionally, the muscle tissue between the posterior surface of the sacrum and skin displayed loss of perfusion on follow-up imaging and was found necrotic upon histopathological examination (Fig. 4c-d). The tissue damage extended more laterally on the left side of the animal.

**Fig. 5:**
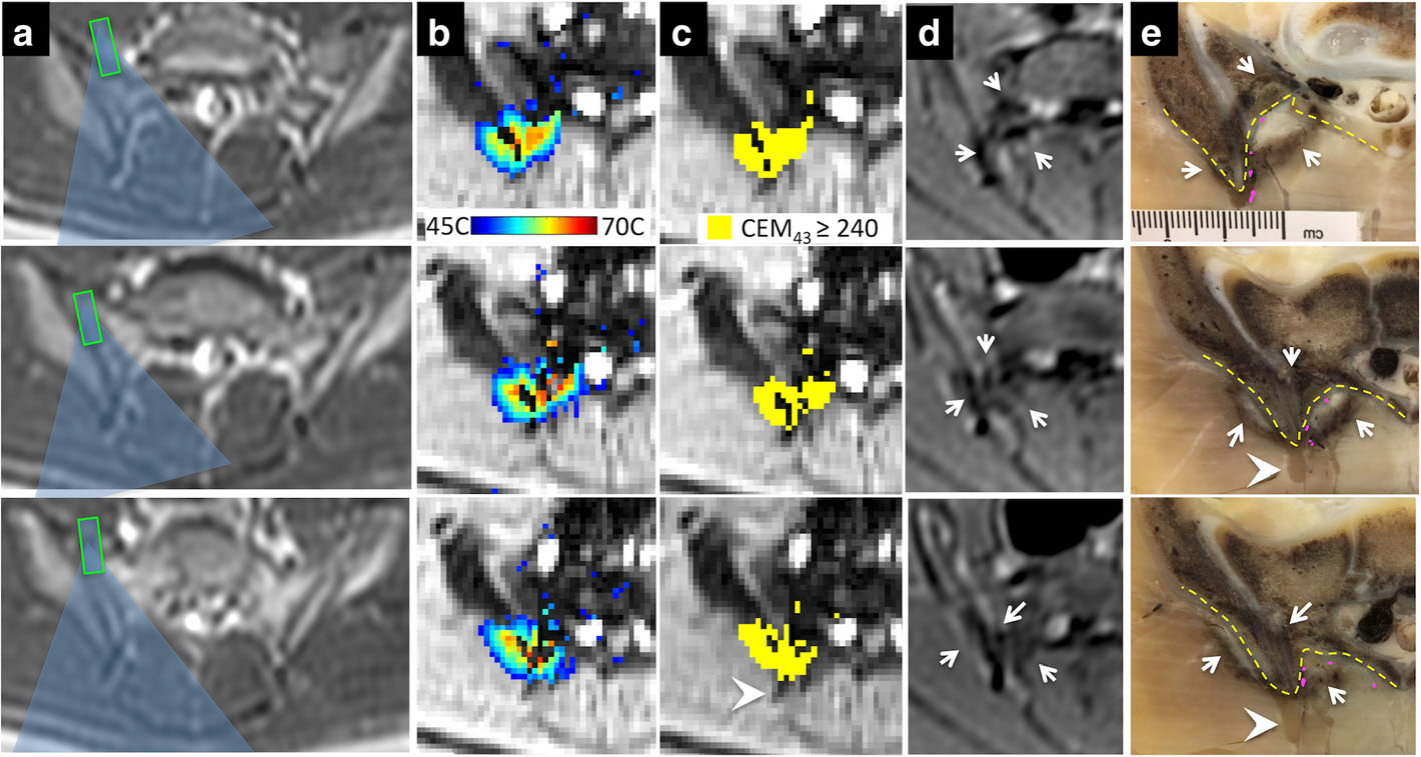
Planning (**a**), monitoring (**b**-**c**) and assessment (**d**-**e**) of the HIFU ablation of the right SIJ in experiment 2. A. Planning of individual sonications is shown as overlay of the axial MR planning images and the approximate HIFU beam path (blue) and focus (rectangle). MR thermometry measurements (**b**) and the resulting thermal dose maps (**c**) obtained during the sonications planned as shown in A. Ablation zone produced during the treatment as visualized on contrast-enhanced MR image obtained immediately following the treatment (**d**) and on gross pathology (**e**). The contours of ablation zone (white arrows) are consistent with the region of perfusion loss seen on MRI and thermal dose map. Dashed line shows the muscle bone interface, the location of the nerves are marked in magenta based on the corresponding histology slides. Arrowheads point to the tip of the sacral ligament

**Fig. 6:**
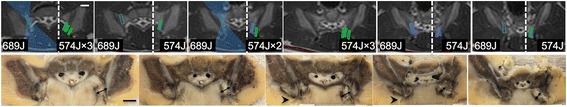
Consecutive slices of gross pathology from the experiment 3 demonstrating ablation zones encompassing the posterior of the left and right SIJ. The upper panel shows the planning images for all sonications performed in this experiment, and the corresponding acoustic energy. Changes in muscle tissue extended further when focal targeting approach was used on the left (black double-sided arrows), compared to the near-field targeting approach used on the right. Ligament tips are shown with black arrowheads

In the subsequent two experiments, the number of sonications was reduced and the cooling time between the sonications was increased (Table 1). In these experiments, the ablated region was well estimated by the thermal dose measurements, derived from MR thermometry (Fig. 5b, c). Perfusion loss encompassed a layer of muscle and superficial layer of sacrum and iliac bones surrounding the SIJ (Fig. 5d). On gross pathology (Fig. 5e), muscle in the treated area appeared pale and hemorrhage, presenting as a brown rim, surrounded the region of ablation. Tissue changes were found penetrating several millimeters into the bone and encompassing several millimeters of the SIJ. In the second and third animal study, the lesions on the left extended further into muscle tissue than on the right, where near-field targeting approach was used (Fig. 6).

**Fig. 7:**
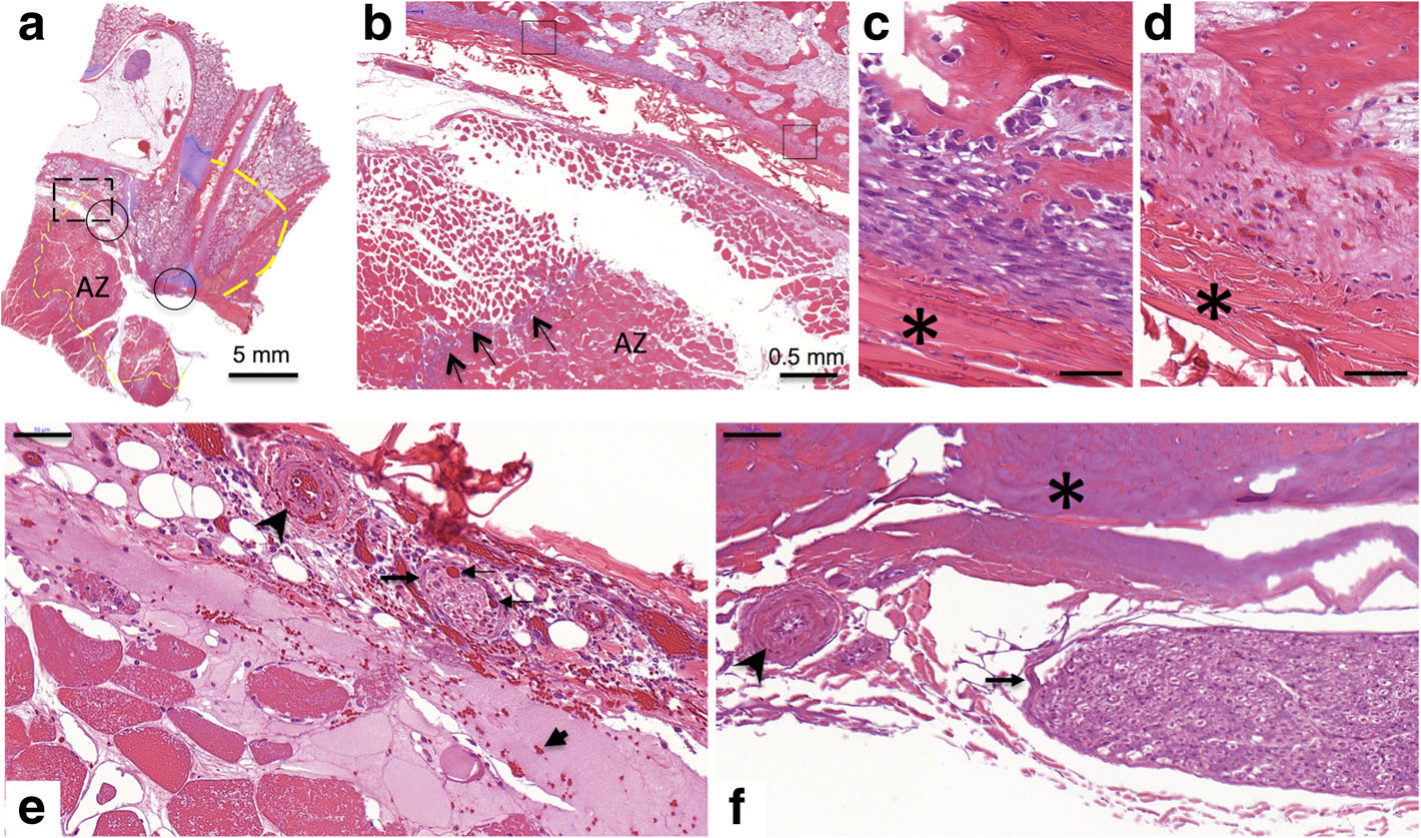
Haematoxylin and eosin histology. **a**. Low magnification image showing ablation zone (AZ) contoured with dashed yellow line. AZ encompasses muscle tissue adjacent to the SIJ. **b**. Boundary of the AZ corresponding to the dashed box in **a**. Accumulation of the macrophages at the boundary is marked with black arrows. **c**. Appearance of the bone surface outside of AZ, corresponding to the solid-line box (left) in **b. d**. Changes in the bone surface caused by HIFU. Asterisk shows the ligament at the surface of the bone. **e**-**f**. High magnification image of the regions marked with the two circles in **a**. Closer to the boundary of AZ the nerve exhibits hyperaemia of endoneurial vessels (think arrows), the stroma surrounding the nerve (thicker arrow) appear pink as in normal nerve. Hemorrhage in the surrounding muscle is marked with a short arrow. The artery (arrowhead) showed fibrinoid necrosis of the tunica media. In the central region of AZ (**f**), the nerve morphology appears unchanged, however, there is pronounced hyalinisation of the collagen in the neural stroma and the ligament tissue on the surface of the bone (asterisk) . The artery (arrowhead) shows hyalinisation of the collagen with the outer surface of the vessel appearing purple compared to pink in **e**. Scale bar is 50 microns

An example of ablation zone contour as determined from the histological analysis is shown on low magnification in Fig. 7. The histological changes found in ablated zone are described below. In muscle, ablation resulted in swelling of fibres, sarcoplasmic hypereosinophilia and fragmentation, and nuclear shrinkage. At the periphery of the muscle lesion, there was accumulation of macrophages and necrosis of the small vessels (Fig. 7b). Bone and bone marrow adjacent to the ablated muscle exhibited changes consistent with coagulative necrosis: cellular shrinkage, cytoplasmic hypereosinophilia and nuclear pyknosis of periosteal osteoblasts and hematopoietic cells, necrotic vessels and hemorrhage (Fig. 7c). Nerves and vessels encompassed by ablation zone displayed two types of changes consistent with necrosis. Near the boundary of the ablation zone (Fig. 7e), the nerve showed hyperemia of endoneurial vessels, nuclear pyknosis of Schwann cells, and loss of axons associated with dilation of the myelin sheath. The color of the ligament on the surface of adjacent bone remained pink. The artery showed fibrinoid necrosis of the tunica media. In the central part of the lesion (Fig. 7f), nerves and vessels exhibited only hyalinization of epineural or adventitial collagen and no structural changes. The ligament on the surface of the bone immediately adjacent to the nerve also showed pronounced hyalinization of the collagen, changing color from pink to purple. The analysis of the spinal cord and nerve root samples showed minor hyperemia in several samples and no changes in others.

### Behavioral assessment

Neither changes in animal behavior and function (ambulation and gait) nor signs of superficial or deep pain were detected during the post-procedural 48-h period.

## Discussion

This work evaluated the potential of MRgHIFU for noninvasive denervation of the SIJ for the first time. The preliminary study presented here demonstrates that MRgHIFU can create contiguous elongated lesions along the SIJ and thermal necrosis of the lateral branch nerves without causing unwanted neurological damage and with minimal thermal damage to the adjacent muscle tissue and bone.

Utilizing swine model for preliminary study of safety and feasibility of HIFU ablation of SIJ is justified by anatomic similarities between porcine and human sacra. The main disadvantage of this model is the smaller size of the lower sacral vertebral bodies compared to those in humans, which makes it challenging to delineate the anatomical landmarks and target the space between the foramina and the SIJ. Nearly nonexistent spinous process in swine enables HIFU beam to be directed at a broad range of angles to reach the target. A more pronounced spinous process bone in humans may limit the angles at which the target can be approached by HIFU.

Oblique orientation of the sacral foramina passages and the small diameter of the posterior foramina compared to that in humans limit direct access to the ventral nerve roots, thus minimizing the risk of any unwanted heating of these critical structures. In humans, the sacrum is flatter and wider leaving more space to position the HIFU lesions between the SIJs and the foramina. At the same time, in human, the larger diameter of the foramina and their orientation toward midline potentially can make the nerve roots more exposed. And while the dorsal nerve root has been directly targeted during RF ablation to treat SIJ pain in the past [35], damage of the ventral nerve roots must be avoided.

Thus, studying the porcine sacral anatomy illustrates the modifications needed for technique regardless of species, and more importantly inter-human variations. It is expected that variations in human anatomy will alter the sonication direction, time, and intensity for appropriate lesioning of the innervation of the SIJ. Understanding the flexibility of MRgHIFU systems will allow a clinician to make appropriate adjustments patient to patient.

Contiguity of the thermal lesions, which is typically challenging to verify during the fluoroscopy-guided RFA, was achieved in all of the experiments as was demonstrated on follow-up contrast-enhanced imaging and histo-pathological analysis. The extent of the lesion in muscle was greater using the focal targeting approach compared to near-field approach. Histology showed thermal damage of the nerves, muscle and bone contained in the targeted area. Histological changes in ablated nerves were consistent with previous work on HIFU ablation of the sciatic nerve in acute rabbit and swine models [24, 27, 36]. While the functional implications of the mild hyperemia observed in several sections of the spinal cord and the nerve roots are not currently known, the lack of any observable changes in animal behavior, gait or function served us as a more meaningful metric for the safety of the treatment.

Unplanned ablation of the muscle region between the sacrum and skin which occurred in the first experiment can be explained by accumulation of the residual heat following each ablation. Thermometry imaging which was performed in axial plane for the most part of treatment on the left side was not capturing the heating during many sonications as was realized upon switching from axial to sagittal thermometry imaging plane. Curvilinear surface of sacrum in cranio-caudal direction led to the heat deposition not necessarily at the position of the planned focal spot, thus single-slice axial plane was not imaging the area being heated during a sonication. Due to this not-immediately realized phenomenon, and high confidence in ablation of a target not previously attempted, a large number of sonications were performed in the first experiment, and the default cooling time was insufficient to ensure the muscle tissue in the near field does not accumulate excessive thermal dose. Furthermore, MR thermometry implemented of the system used here computes the temperature increase during a sonication relative to the temperature reference in the beginning of each sonication, which may lead to underestimation of thermal dose [37].

Measures taken in the other two experiments, i.e. reduction of the number of sonications, increase of the cooling time and frequent use of sagittal MR thermometry plane, showed that SIJs can be safely ablated without excessive near-field heating. Fortunately, newer version of the clinical MRgHIFU systems offer multi-slice thermometry capability, which will enable capturing the highest temperatures during sonications even when it is not positioned at the expected location. Additionally, long-term MR thermometry, proposed by R. Bitton in [37], should help monitor temperature accumulation in muscle near bone thus enabling optimization of the cooling time.

While the initial results presented in this article show promise for MRgHIFU in non-invasive and efficient treatment of SIJ, further studies are needed to optimize this procedure. Focusing approach, the number of sonications and the energy range explored here were effective in creating thermal lesions, however, they can be further refined to reduce the procedure time and to minimize thermal damage in bone and muscle. While pre-focal targeting approach, which was applied to the right SIJs in this study, can help reduce the procedure time owing to the larger ablated surface per sonication, the opening of the dorsal foramina needs to be carefully avoided affecting the area that can be safely treated in one sonication. Proximity of skin to the SIJ needs to be taken into account to avoid unwanted heating of the skin and adjacent muscle tissue. Cooling time between the subsequent sonications is the parameter that will need to be optimized in order to provide sufficient time for the skin to cool off while keeping the treatment time minimal. As accurate MR thermometry is challenging in the bone, in this study, we monitored the changes in muscle temperature during each sonication.

One of the limitations of this study is the lack of measurements of nerve conduction, which could confirm whether HIFU ablation indeed denervated the SIJs. Unfortunately due to location of the targeted nerves, deep inside the muscle tissue immediately adjacent to the bone and their relatively small size, nerve conduction studies were not feasible. Therefore, disruption of the nerves in this study is currently implied from the presence of the thermal lesions encompassing the nerves and the thermal changes of the nerve.

## Conclusions

This preliminary study of MRgHIFU ablation of SIJs in sub-acute swine model shows promise for this ablation modality as a non-invasive potentially more precise alternative to the currently used fluoroscopic-guided RFA and injections. This current research is a proof of concept for the use of HIFU for denervation of the SIJ in the human population. This preliminary proof-of-concept experiment offers initial guidance to subsequent animal, cadaver and clinical studies. Further study is warranted to determine appropriate treatment protocol for MRgHIFU of the lateral branches in the human population.

## Abbreviations

CE-CT: Contrast-enhanced computerized tomography
CE-MRI: Contrast-enhanced magnetic resonance imaging
CT: Computerized tomography
MRgHIFU: Magnetic resonance imaging guided high intensity focused ultrasound
MRI: Magnetic resonance imaging
RF: Radio-frequency
RFA: Radio-frequency ablation
SIJ: Sacroiliac joint.
